# Analytical and functional similarity of Amgen biosimilar ABP 215 to bevacizumab

**DOI:** 10.1080/19420862.2018.1452580

**Published:** 2018-04-20

**Authors:** Neungseon Seo, Alla Polozova, Mingxuan Zhang, Zachary Yates, Shawn Cao, Huimin Li, Scott Kuhns, Gwendolyn Maher, Helen J. McBride, Jennifer Liu

**Affiliations:** a Biosimilars, Amgen Inc., One Amgen Center Drive, Thousand Oaks, CA, USA; b Process Development, Amgen Inc., Cambridge, MA, USA; c Process Development, Amgen Inc., One Amgen Center Drive, Thousand Oaks, CA, USA

**Keywords:** ABP 215, bevacizumab, biosimilar, analytical similarity assessment, structure, function

## Abstract

ABP 215 is a biosimilar product to bevacizumab. Bevacizumab acts by binding to vascular endothelial growth factor A, inhibiting endothelial cell proliferation and new blood vessel formation, thereby leading to tumor vasculature normalization. The ABP 215 analytical similarity assessment was designed to assess the structural and functional similarity of ABP 215 and bevacizumab sourced from both the United States (US) and the European Union (EU). Similarity assessment was also made between the US- and EU-sourced bevacizumab to assess the similarity between the two products. The physicochemical properties and structural similarity of ABP 215 and bevacizumab were characterized using sensitive state-of-the-art analytical techniques capable of detecting small differences in product attributes. ABP 215 has the same amino acid sequence and exhibits similar post-translational modification profiles compared to bevacizumab. The functional similarity assessment employed orthogonal assays designed to interrogate all expected biological activities, including those known to affect the mechanisms of action for ABP 215 and bevacizumab. More than 20 batches of bevacizumab (US) and bevacizumab (EU), and 13 batches of ABP 215 representing unique drug substance lots were assessed for similarity. The large dataset allows meaningful comparisons and garners confidence in the overall conclusion for the analytical similarity assessment of ABP 215 to both US- and EU-sourced bevacizumab. The structural and purity attributes, and biological properties of ABP 215 are demonstrated to be highly similar to those of bevacizumab.

## Introduction

ABP 215 (US: MVASI™ [bevacizumab-awwb] approved in September 2017; EU: MVASI™ [bevacizumab] approved in January 2018; Amgen Inc.) is a biosimilar to bevacizumab (Avastin®), a recombinant humanized monoclonal immunoglobulin G1 (IgG1) antibody. Bevacizumab binds to vascular endothelial growth factor A (VEGF-A) and prevents the binding of VEGF-A to VEGF receptors on the surface of endothelial cells, inhibiting endothelial cell proliferation and new blood vessel formation, thereby leading to normalization of the tumor vasculature.^1^ Bevacizumab was first approved by the United States Food and Drug Administration (FDA) in 2004, and then by the European Medicines Agency (EMA) in 2005, to treat patients with certain types of cancers where tumor vasculature contributes to tumor growth.^2^

ABP 215 is the first approved biosimilar to bevacizumab in the US and EU. ABP 215 is approved in the US for the treatment of metastatic colorectal cancer, non-squamous non–small cell lung cancer, glioblastoma, metastatic renal cell carcinoma, and persistent, recurrent, or metastatic carcinoma of the cervix.^3^ In the EU, ABP 215 is approved for the treatment of metastatic carcinoma of the colon or rectum, metastatic breast cancer, non-squamous non–small cell lung cancer, metastatic renal cell cancer, ovarian cancer, fallopian tube or primary peritoneal cancer, and persistent, recurrent, or metastatic carcinoma of the cervix.^4^

Biosimilars have the potential to bring increased access to important therapeutics to a broader patient population. However, transforming complex therapeutic proteins into effective biological medicines requires highly specialized knowledge and experience with scientific standards, processes, and quality systems. Since each biosimilar product must establish a unique cell line and manufacturing process, biosimilars are not expected to be identical to their reference products. Instead, biosimilars will have minor differences in product attributes, which do not impact clinical safety and efficacy. The FDA and EMA provide guidelines for generation of a robust data package of analytical, biological, non-clinical and clinical data to demonstrate similarity of structure and function with no notable risks to the safety and efficacy of the product in clinical use.^5-7^

Here, we present the plan and results of a comprehensive analytical similarity assessment of Amgen's biosimilar ABP 215 to assess its analytical similarity with bevacizumab. The results also include a comparison of bevacizumab from the US (bevacizumab [US]) and European Union (bevacizumab [EU]). The analytical similarity assessment plan was designed to assess structural/physicochemical and functional similarity and ensure the understanding of whether any differences between ABP 215 and bevacizumab had the potential to impact clinical performance, consistent with US and EU regulatory guidelines.^5-7^

## Results

The testing plan listing all the analytical techniques and attributes/assays evaluated for ABP 215 and bevacizumab is shown in Table 1. The purpose of the plan was to evaluate both active ingredients and inactive ingredients that could affect product safety and efficacy in addition to product quality. Where applicable, orthogonal methods were used to fully analyze product attributes and activities.

**Table 1.** Similarity testing plan and the analytical methods for the structural and functional characterization of the proposed biosimilar ABP 215 and bevacizumab reference products.

Category	Analytical Technique
Primary Structure	Molecular mass of intact whole protein
	Molecular mass of reduced and deglycosylated HC and LC
	Protein sequence by reduced peptide map
	Disulfide structure by non-reduced peptide map
	N-glycan map by HILIC HPLC
	Extinction coefficient by amino acid analysis
	Isoelectric point by cIEF
	Identity by anti-idiotype ELISA
Higher Order Structure	Secondary structure by FTIR
	Tertiary structure by near UV-CD
	Thermal stability by DSC
Particles and Aggregates	Subvisible particles by light obscuration
	Subvisible particles by MFI
	Submicron particle profile by DLS
	Submicron particle profile by FFF
	Aggregates by AUC-SV
	Aggregates by SE-HPLC-LS
Product-related Substances and Impurities	Size variants by SE-HPLC, rCE-SDS and nrCE-SDS
	Charge variants by CEX-HPLC
Thermal Forced Degradation	Thermal stability at 25, 40, and 50°C assessed by purity and potency
Biological Activity	VEGF-A binding (ELISA)
	Proliferation inhibition bioassay (potency)
	VEGF-A binding kinetics and affinity
	Binding to VEGF-A isoforms
	Inhibition of VEGFR-2 RTK autophosphorylation
	Specificity by VEGFR-2 RTK autophosphorylation
	FcRn binding
	FcγRIa binding
	FcγRIIa binding
	FcγRIIb binding
	FcγRIIIa (158 V) binding
	FcγRIIIa (158 F) binding
	FcγRIIIb binding
	C1q binding
	Lack of ADCC activity
	Lack of CDC activity
General Properties	Protein concentration and volume
	Osmolality, pH, appearance, color, and clarity
Process-Related Impurities	HCP by ELISA, 2D LC-MS, and 2D-DIGE
	Residual protein A by ELISA
	Residual DNA analysis by qPCR

Abbreviations: 2D-DIGE = 2-dimensional differential in-gel electrophoresis, 2D LC-MS = 2-dimensional liquid chromatography coupled with online mass spectrometry involving data-independent MS acquisition, ADCC = antibody-dependent cell-mediated cytotoxicity, AUC-SV = analytical ultracentrifugation sedimentation velocity, CDC = complement-dependent cytotoxicity, CEX-HPLC = cation exchange high performance liquid chromatography, cIEF = capillary isoelectric focusing, C1q = the first subcomponent of the C1 complex of the classical pathway of complement activation, DSC = differential scanning calorimetry, ELISA = enzyme-linked immunosorbent assay, FcR = fragment crystallizable receptor, FcγRIa = Fc gamma receptor Type Ia, FcγRIIa = Fc gamma receptor Type IIa, FcγRIIb = Fc gamma receptor Type IIb, FcγRIIIa = Fc gamma receptor Type IIIa, FcγRIIIb = Fc gamma receptor Type IIIb, FcRn = neonatal Fc receptor, FFF = field flow fractionation, FTIR = Fourier-transform infrared spectroscopy, HC = heavy chain, HCP = host cell protein, HILIC = hydrophilic interaction liquid chromatography, HPLC = high performance liquid chromatography, LC = light chain, MFI = micro-flow imaging, nrCE-SDS = non-reduced capillary electrophoresis – sodium dodecyl sulfate, qPCR = quantitative polymerase chain reaction, rCE-SDS = reduced capillary electrophoresis – sodium dodecyl sulfate, SE-HPLC-LS = size exclusion high performance liquid chromatography with light scattering, SE-HPLC = size exclusion high performance liquid chromatography, UV-CD = ultraviolet circular dichroism, VEGF-A = vascular endothelial growth factor A.

More than 20 batches of US bevacizumab and EU bevacizumab were tested over six years. The ABP 215 batches used in similarity assessment represent drug product lots filled from 13 individual drug substance lots manufactured over the same period. At least 13 ABP 215 lots were tested for product attributes such as purity and glycan structure, which could be affected by the drug substance manufacturing process. For product attributes that could be influenced by fill–and-finish process, including volume and particles, all drug product batches were tested for similarity. This testing plan resulted in a large number of datasets, which enabled meaningful comparisons, including the use of statistical analysis per regulatory agency guidance, and helped further garner confidence in the overall conclusion for the analytical similarity assessment of ABP 215 to both US- and EU-sourced bevacizumab.

### Primary structure

To investigate the analytical similarity of the primary structures of ABP 215 and bevacizumab, several complementary characterization methods were used, employing state-of-the-art analytical tools, such as high precision and accuracy mass spectrometry for protein sequence, and high-resolution chromatography with sensitive detection for oligosaccharide profiles.

**Figure 1.**
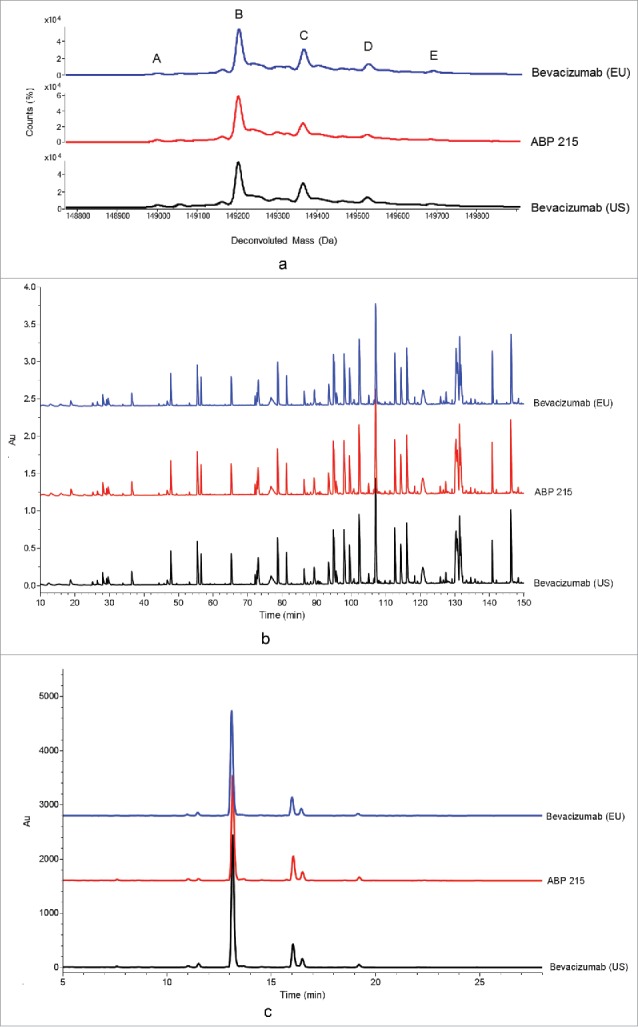
Primary Structure: Comparison of ABP 215, Bevacizumab (US), and Bevacizumab (EU) Intact Molecular Mass Profile (a), reduced peptide map (b), and glycan map (c).

Results of intact mass analysis, reduced peptide map, and glycan map are shown in Fig. 1a-c, respectively. As shown in Fig. 1a, the predominant species are consistent with the presence of two core-fucosylated complex N-linked glycans with either 0, 1, or 2 terminal galactose residues, depicted as A2G0F, A2G1F, and A2G2F, respectively. Peaks A, B, C, D, and E were identified as the predominant forms with molecular weights consistent with a structure that contains two glycans composed of A2G0F:A1G0F (peak A); A2G0F:A2G0F (peak B); A2G0F:A2G1F (peak C); A2G1F:A2G1F or A2G0F:A2G2F (peak D); and A2G1F:A2G2F (peak E). The intact molecular mass of all predominant species matched the theoretical mass for the products (Table 2).

**Table 2.** Summary of attributes for ABP 215, bevacizumab (US), and bevacizumab (EU).

Analytical Testing/Attributes	ABP 215 Range (n)	Bevacizumab US Range (n)	Bevacizumab EU Range (n)
Intact molecular weight (Da)			
A: Glycosylation – A2G0F:A1G0F	149008–149017 (4)	149007–149015 (4)	149009–149025 (4)
B: Glycosylation – A2G0F:A2G0F	149210–149219 (4)	149210–149218 (4)	149209–149218 (4)
C: Glycosylation – A2G0F:A2G1F	149371–149381 (4)	149374–149378 (4)	149373–149381 (4)
D: Glycosylation – A2G1F:A2G1F or A2G0F:A2G2F	149536–149541 (4)	149531–149535 (4)	149531–149538 (4)
E: Glycosylation – A2G1F:A2G2F	149694–149698 (4)	149673–149698 (4)	149663–149710 (4)
Reduced and deglycosylated heavy chain (Da)	49744–49752 (4)	49741–49753 (4)	49742–49753 (4)
Reduced and deglycosylated light chain (Da)	23479–23482 (4)	23479–23481 (4)	23479–23482 (4)
Glycosylation at Asn_303_ (%)	99.2–99.4 (13)	97.9–98.3 (24)	97.9–98.4 (26)
Glycan map (%)			
High mannose	1.2–2.7 (13)	0.3–1.3 (23)	0.5–1.2 (25)
Afucosylation	1.2–1.7 (13)	1.7–3.8 (23)	1.9–2.6 (25)
Galactosylation	17.1–29.4 (13)	8.7–21.8 (23)	7.8–21.2 (25)
Sialic acid	0.2–0.3 (13)	0.1–0.2 (23)	0.1–0.2 (25)
FTIR/spectral similarity (%)			
US RP	97.6–99.8 (6)	97.9–99.3 (6)	97.2–99.0 (6)
EU RP	97.9–99.8 (6)	98.3–99.7 (6)	97.5–99.3 (6)
Near UV-CD/spectral similarity (%)			
US RP	98.5–99.4 (6)	98.5–99.6 (6)	97.3–99.4 (6)
EU RP	97.1–99.4 (6)	97.0–99.6 (6)	97.6–99.5 (6)
DSC (°C)			
T_m1_	72.7–73.1 (11)	72.6–73.1 (12)	72.5–73.1 (12)
T_m2_	83.2–83.7 (11)	83.2–83.9 (12)	83.0–84.0 (12)
LO subvisible particle (particles/container)			
≥ 2 µm	320–5736 (19)	48–4749 (14)	77–15493 (15)
≥ 5 µm	48–1488 (19)	14–755 (14)	24–688 (15)
≥ 10 µm	16–219 (19)	3–112 (14)	8–224 (15)
≥ 25 µm	0–96 (19)	0–32 (14)	0–32 (15)
MFI subvisible particle (particles/mL)			
≥ 5 µm spherical particles	16–218 (19)	42–3439 (11)	51–588 (12)
≥ 5 µm non-spherical particles	0–65 (19)	6–2157 (11)	8–430 (12)
AUC-SV/monomer (%)	98.0–99.3 (6)	97.3–98.6 (6)	97.3–98.6 (6)
SE-HPLC-LS MW (kDa)			
Monomer	148 (3)	148 (3)	148 (3)
Dimer	297–304 (3)	300–301 (3)	294–298 (3)
Protein concentration (mg/mL)	24.4–25.8 (13)	23.9–25.9 (24)	24.5–25.8 (26)
Volume (mL)			
100 mg/4 mL	4.1–4.3 (7)	4.2–4.3 (6)	4.3–4.7 (5)
400 mg/16 mL	16.3–16.5 (12)	16.2–16.6 (6)	16.3–16.8 (8)
CHO cell protein by ELISA (ng/mg)	4–12 (13)	6 (3)	5–6 (3)

n indicates a number of samples tested

The molecular masses of reduced and deglycosylated drug product provided further evidence that the heavy chain (HC) and light chain (LC) polypeptide compositions were similar between ABP 215 and bevacizumab (Table 2). The observed masses for both ABP 215 and bevacizumab match closely to the theoretical masses calculated based on the coding oligonucleotide sequences, including the conversion of Asn_303_ to Asp_303_ as a result of removal of the N-glycans by PNGase F and the absence of the C-terminal lysine on the HC. The major peaks correspond to the expected theoretical masses of reduced and deglycosylated HC and LC. In addition, a minor peak with a mass addition of 162 Daltons corresponding to glycated HC and LC were present in both ABP 215 and bevacizumab. Glycation is a type of chemical modification, which takes place primarily during the cell culture process, and most commonly affects the *e*-amino side chain of lysine residues and N-terminal amines of a protein.^8^ Protein glycation occurs under physiological conditions and the typical low levels of glycation found in monoclonal antibodies are not expected to have any safety impacts.^8^ The levels of glycation observed in both HC and LC for ABP 215 and bevacizumab were all low and comparable.

The reduced peptide map overlays (Fig. 1b) showed similar peak profiles for ABP 215 and bevacizumab, and the differences between the observed and theoretical masses for all tryptic peptides were within the specified mass accuracy based on instrument and method capability. The same post-translational modifications were detected in both ABP 215 and bevacizumab, and no new species were detected. Examination of the amino acid sequence revealed a single consensus site for N-linked glycosylation located on the HC at Asn_303_ in all ABP 215 and bevacizumab peptide maps. LC-MS data showed the prevalent glycan structures to be core fucosylated biantennary complex structures with 0 or 1 terminal galactose, consistent with the glycan map results, as discussed in this section.

Several low abundance peptides were observed in both ABP 215 and bevacizumab. Characterization by mass spectrometry identified the modifications, which included non-glycosylation at Asn_303_ site, cyclization of the HC N-terminal Glu_1_ residue to pyroglutamate (pE), truncation of the HC C-terminal Lys_453_ residue, and a few deamidation and oxidation sites. The same modifications and sites were observed in both ABP 215 and bevacizumab, and the levels of these post-translational modifications were similar.

In addition, the non-reduced peptide map confirmed that both ABP 215 and bevacizumab contain a total of 32 cysteine residues comprising 11 cysteines in each HC and 5 cysteines in each LC. Under native conditions, these residues form a total of 16 disulfide bonds, including 12 intrachain and 4 interchain disulfide bonds. The presence of the expected 16 cysteine-containing residues was confirmed for both ABP 215 and bevacizumab. The difference between the observed molecular masses and the theoretical masses for the disulfide-linked peptides in the non-reduced peptide maps, and the cysteine-containing peptides in the reduced peptide maps, were all within the specified mass accuracy based on instrument and method capability.

The overlay of glycan maps for ABP 215 and bevacizumab (US and EU) is shown in Fig. 1c. The results demonstrate ABP 215 and bevacizumab have similar profiles with a total of 19 glycan forms, and all major glycans are present in both ABP 215 and bevacizumab. As shown in Table 2, four glycan groups were evaluated as part of the similarity assessment based on their potential to affect pharmacokinetics (PK) or biological functions, including binding to crystallizable fragment (Fc) receptors. The minor differences at these observed levels do not affect any of the biological activities, as shown in Table 3.

**Table 3.** Summary of functional attributes for ABP 215, bevacizumab (US), and bevacizumab (EU).

Analytical Testing/Attributes	ABP 215 Range (n)	Bevacizumab US Range (n)	Bevacizumab EU Range (n)
Inhibition of proliferation (potency) (%)	91–105 (13)	86–104 (24)	88–103 (25)
Relative VEGF-A binding (%)	84–98 (13)	80–104 (14)	76–99 (13)
VEGF-A binding affinity (K_D_)	20.1–23.5 (3)	22.9–23.9 (3)	18.4–23.4 (3)
Binding to FcRn (%)	84–104 (13)	91–107 (14)	88–103 (14)
Binding to FcγRIa (%)	90–98 (3)	83–90 (3)	99–102 (3)
Binding to FcγRIIa (%)	83–101 (3)	89–91 (3)	80–90 (3)
Binding to FcγRIIb (%)	92–98 (3)	90–94 (3)	87–91 (3)
Binding to FcγRIIIa, 158V (%)	78–115 (13)	77–98 (12)	81–117 (17)
Binding to FcγRIIIa, 158F (%)	84–120 (13)	80–110 (10)	83–109 (10)
Binding to FcγRIIIb (%)	89–92 (3)	80–85 (3)	67–88 (3)
Binding to C1q (%)	91–122 (13)	88–115 (10)	90–104 (9)

n indicates a number of samples tested

High-mannose glycan forms, including M5, M6, M7, and M8, have the potential to affect PK via differential clearance through binding to mannose-binding receptors.^9^ In addition, they could influence antibody-dependent cell-mediated cytotoxicity (ADCC) via changes in binding to FcγRIIIa.^10^ However, due to the soluble nature of the target, ABP 215 and bevacizumab do not exhibit ADCC activity.^11^ ABP 215 had slightly higher levels of high mannose (Table 2), but the minor differences at the levels observed have no effect on FcγRIIIa binding activity (Table 3) and are not considered clinically meaningful since ABP 215 has a similar PK profile compared with bevacizumab.^9^^,^^12^

Afucosylated glycan forms, N-linked glycans that lack core fucose, include complex-type, hybrid-type, and high mannose glycans.^12^ Afucosylation of the N-linked glycan of the Fc region can enhance FcγRIIIa binding of IgG1 antibodies.^10^ ABP 215 had slightly lower levels of afucosylation (Table 2), but this minor difference at the level observed has no impact on FcγRIIIa binding activity (Table 3) and is not considered clinically meaningful since ABP 215 and bevacizumab do not exhibit ADCC activity.^11^

Galactosylated glycan forms include all complex and hybrid glycan structures that contain at least one terminal galactose.^12^ Galactosylation has the potential to affect binding to C1q, which is a key step in the activation of IgG1-mediated cell lysis via complement-dependent cytotoxicity (CDC).^13^^,^^14^ ABP 215 had slightly higher levels of galactosylation (Table 2), but the minor difference at the level observed has no impact on C1q binding activity (Table 3) and is not considered clinically meaningful since ABP 215 and bevacizumab do not exhibit CDC due to the soluble nature of the target.^11^

Sialylated glycan forms include all complex and hybrid glycan structures that contain at least one terminal sialic acid.^15^ Both ABP 215 and bevacizumab have similarly low levels of sialyation at or near the limit of quantitation (0.1%) of the assay (Table 2), which have no biological impact as demonstrated by the results of the functional similarity assessment.

**Figure 2.**
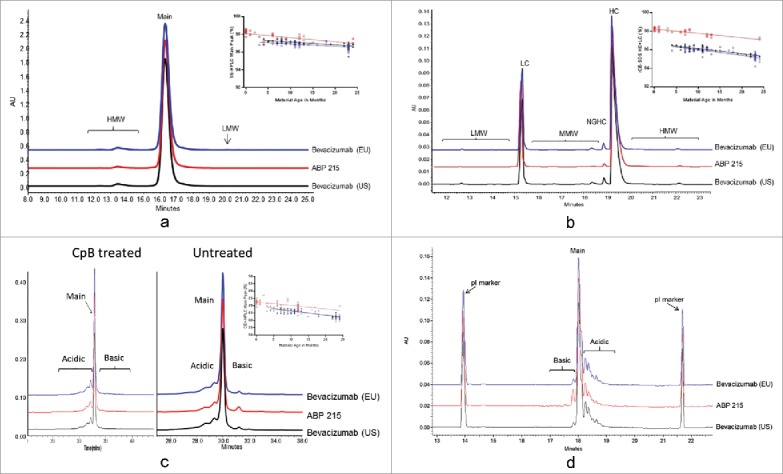
Physicochemical Properties of Size and Charge Variants, as Assessed by SE-HPLC (a), rCE-SDS (b), CEX-HPLC (c), and cIEF (d) for ABP 215 (□), bevacizumab (US) (∆), and bevacizumab (EU) (○).

The apparent isoelectric point (pI) of the protein is influenced by the amino acid sequence and its higher order structure. The apparent pI evaluated using capillary isoelectric focusing (cIEF) showed that the charge distribution profiles for ABP 215 and bevacizumab were similar, and no new peaks were observed (Fig. 2d).

### Higher order structure

The higher order structure of ABP 215 and bevacizumab was characterized using multiple biophysical techniques.

**Figure 3.**
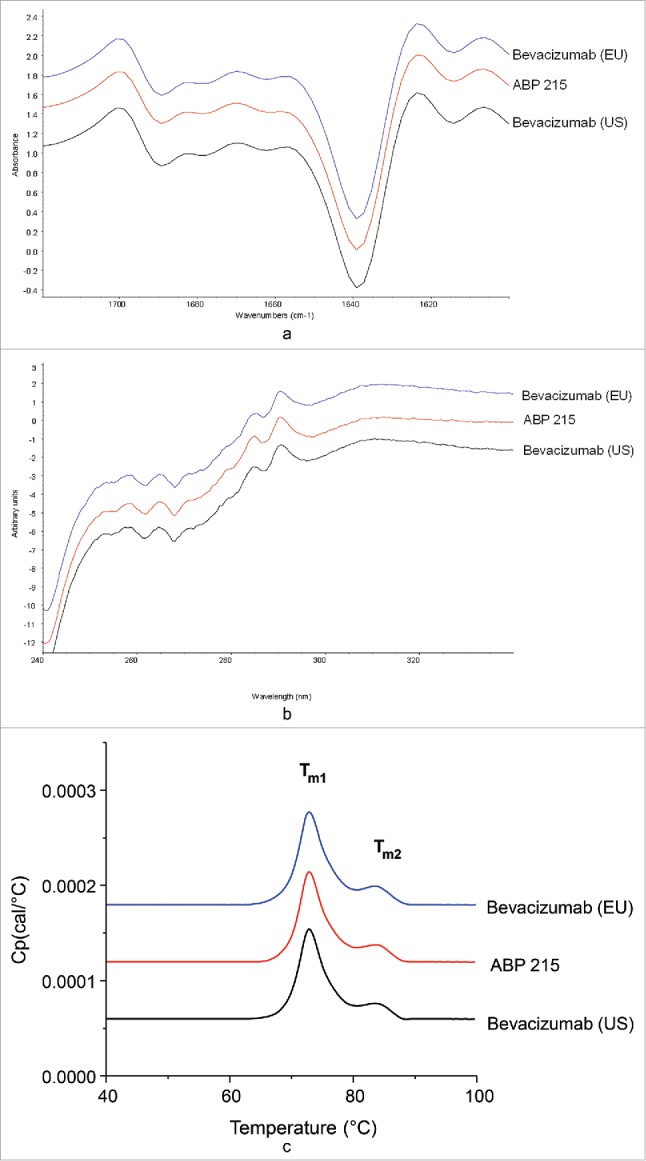
Higher Order Structure: Comparison of ABP 215, Bevacizumab (US), and Bevacizumab (EU) Fourier-transform infrared spectra (a), ultraviolet circular dichroism spectra (b), and differential scanning calorimetry thermograms (c).

Results of secondary structure by Fourier-transform infrared spectroscopy (FTIR), tertiary structure by ultraviolet circular dichroism (UV-CD), and thermal stability by differential scanning calorimetry (DSC) are shown in Fig. 3a-c, respectively. The FTIR profiles were visually similar between ABP 215 and bevacizumab (Fig. 3a). The spectra exhibit strong β-sheet bands at around 1639 cm^−1^ and at 1689 cm^−1^, indicating the presence of antiparallel β-sheet structure existing in typical antibodies. The spectral similarity values of ABP 215 and bevacizumab are ≥ 95% (Table 2), with 95% being the limit of the method's precision. The secondary structures of ABP 215 and bevacizumab were demonstrated to be similar.

The near UV-CD profiles were visually similar between ABP 215 and bevacizumab (Fig. 3b). The spectra contain signals from tryptophan, tyrosine, and phenylalanine, superimposed on the broad disulfide signal from 250 to 280 nm, indicating the presence of native tertiary structure and that the disulfide bonds and aromatic amino acids are in the expected environment due to proper folding of the proteins. The spectral similarity values of ABP 215 and bevacizumab are ≥ 95% (Table 2), with 95% being the limit of the method's precision. The tertiary structure of ABP 215 and bevacizumab is demonstrated to be similar.

When visually compared, the DSC profiles of ABP 215 and bevacizumab were similar (Fig. 3c). The DSC profiles have two endothermic thermal transitions corresponding to the unfolding of the antigen-binding fragment (Fab)/C_H_2 and C_H_3 domains, as characterized by the DSC thermal melting temperatures (T_m_). The T_m1_ and T_m2_ values of ABP 215 and bevacizumab were similar, as summarized in Table 2. The thermal stabilities of ABP 215 and bevacizumab were demonstrated to be similar.

### Particles and aggregates

**Figure 4.**
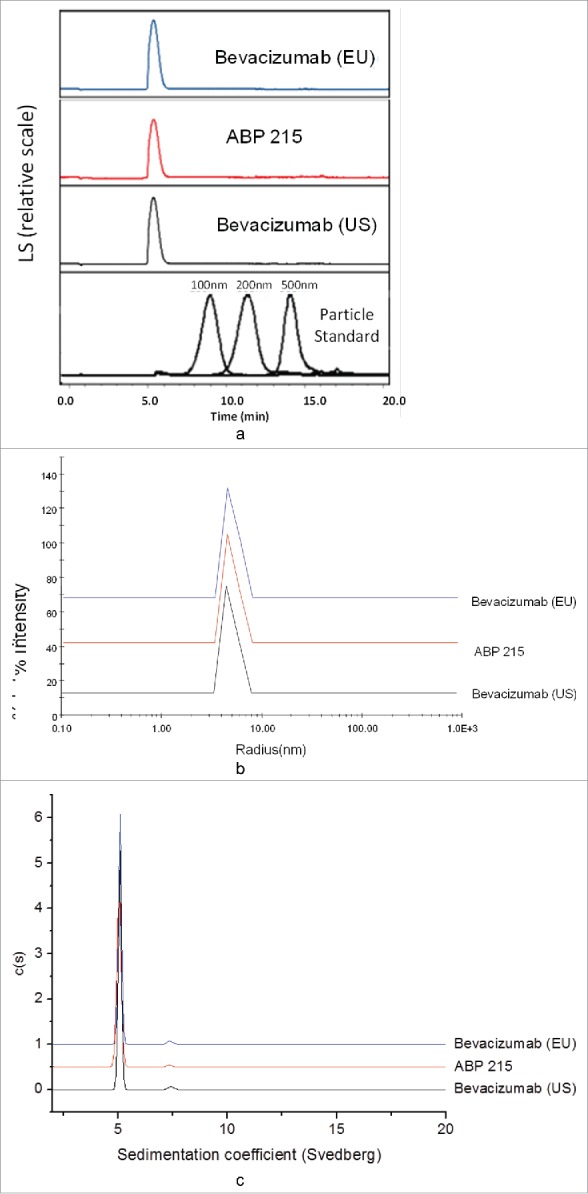
Particles and Aggregates: Comparison of ABP 215, Bevacizumab (US), and Bevacizumab (EU) submicron particle profiles by Field Flow Fractionation (a), dynamic light scattering (b), and aggregates by analytical ultracentrifugation-sedimentation velocity (c).

ABP 215 has similarly low levels of subvisible particles compared with bevacizumab as assessed by light obstruction (LO) and micro-flow imaging (MFI) (Table 2). No submicron particles above the sensitivity of the assay were detected for any of the products as assessed by field flow fractionation (FFF) and dynamic light scattering (Fig. 4a and 4b). Aggregates assessed by AUC-SV (Fig. 4c and Table 2) and SE-HPLC-LS (Table 2) were similar between ABP 215 and bevacizumab. These results demonstrate that ABP 215 has similar particle and aggregate levels compared with bevacizumab.

### Product-related substances and impurities

Product-related substances and impurities of ABP 215, bevacizumab (US), and bevacizumab (EU) were assessed using a combination of methods that evaluate size and charge variants. ABP 215 has a slightly lower level of aggregates, as shown in SE-HPLC (Fig. 2a), and non-glycosylated HC, as shown in reduced CE-SDS (Fig. 2b), compared with bevacizumab.

Assessment of charge variants by CEX-HPLC and cIEF showed that ABP 215 has a slightly higher level of basic variants due to unprocessed HC C-terminal lysine compared with bevacizumab. This was further confirmed by treatment with carboxypeptidase B (CpB), which showed the basic peaks were reduced to similar levels after CpB treatment for all three products (Fig. 2c). cIEF data supported the conclusion that the same charged species are present in all three products (Fig. 2d), with higher level of basic peak corresponding to unprocessed HC C-terminal lysine. Further characterization of the charge variants by peptide mapping showed the same modifications present in both ABP 215 and bevacizumab, and the minor quantitative differences observed does not affect biological activity (Table 3).

Size and charge variants are stability-indicating attributes, which may change as a function of storage time at the recommended storage condition. Therefore, any similarity assessment of the quantitative levels of these product-related variants requires consideration of material age at the time of analysis. The results for the product-related variants were plotted against the estimated material age at the time of testing. ABP 215 and bevacizumab have similar degradation rates for the size and charge variants, as shown in Fig. 2a for SE-HPLC main peak, Fig. 2b for reduced CE-SDS purity (HC plus LC), and Fig. 2c for CEX-HPLC main peak.

### Stability and degradation at accelerated conditions

**Figure 5.**
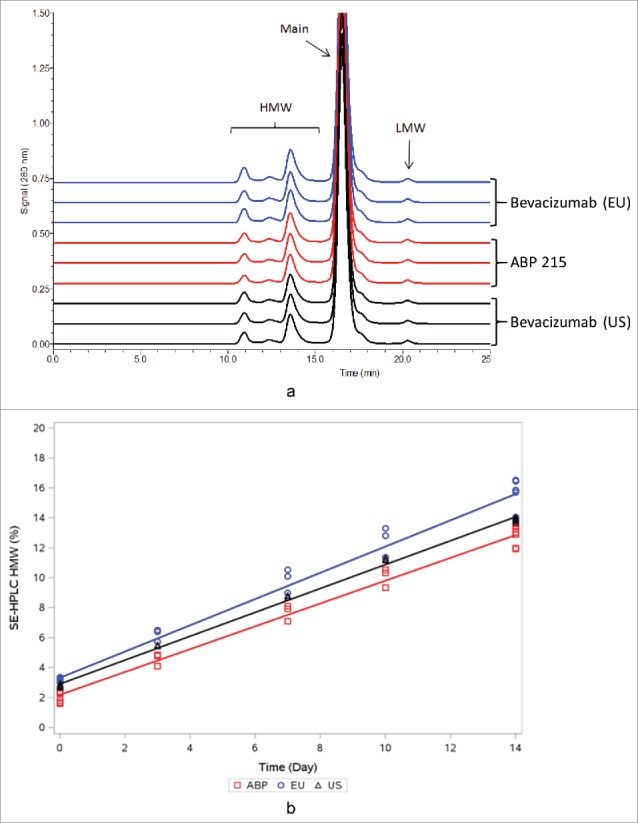
Size Exclusion High Performance Liquid Chromatography Profiles of ABP 215 and Bevacizumab Incubated at 50C for 14 days.

As part of the analytical similarity assessment, thermal stability and degradation studies were performed at 25°C, 40°C, and 50°C to aid in the comparison of product degradation pathways, which could be influenced by residual impurity from manufacturing process. Changes in the stability-indicating attributes were assessed using SE-HPLC, reduced CE-SDS, CEX-HPLC, and potency methods. Degradation profile results for thermally degraded test samples after incubation at 50°C for 14 days were shown to be similar between ABP 215 and bevacizumab for both the presence and the absence of degraded species, as illustrated for SE-HPLC (Fig. 5a). Similarity was further demonstrated by plotting degradation rates for all samples' stability-indicating attributes at the designated intervals, as shown for SE-HPLC main peak at 50°C for up to 14 days (Fig. 5b). The degradation rates for ABP 215 and bevacizumab for all evaluated product-related variants and potency at 25°C, 40°C, and 50°C conditions were found to be similar.

### Biological and functional activity

The biological and functional activities of ABP 215 and bevacizumab evaluated in the similarity assessment are listed in Table 1. Both Fab-mediated binding and functional activities, as well as Fc-mediated binding activities, were assessed. Since ABP 215 and bevacizumab do not exhibit effector functions,^11^ the Fc-mediated binding activities were included to characterize the higher order structure of the molecules. The results from assays reporting quantitative data from the biological and functional assessment are summarized in Table 3.

**Figure 6.**
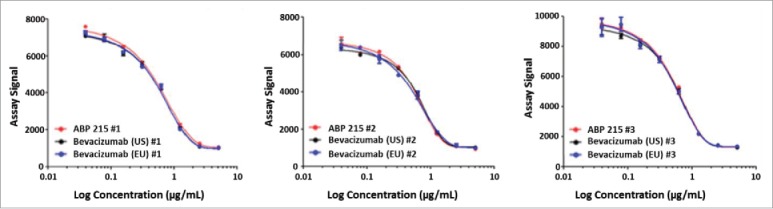
Inhibition of VEGFR-2 RTK autophosphorylation by ABP 215, bevacizumab (US), and bevacizumab (EU).

The biological functions that contribute to the clinical efficacy of bevacizumab are mediated by the neutralization of VEGF-A through the Fab domain. The similarity assessment included potency as measured by inhibition of proliferation in human umbilical vein endothelial cells (HUVEC) and relative binding to the target (VEGF-A). The results demonstrate that ABP 215 has similar potency and VEGF-A binding activity compared with bevacizumab. In addition, side-by-side comparison of binding affinity for VEGF-A further supports that ABP 215 has similar VEGF-A binding activity compared to bevacizumab. Furthermore, the blockade of signaling downstream of VEGF receptor 2 (VEGFR-2), the primary functional receptor for VEGF-A, was evaluated using a receptor tyrosine kinase (RTK) assay in HUVEC. The results support that ABP 215 demonstrates similar inhibition of VEGFR-2 signaling compared with bevacizumab (Figure 6).

VEGF-A acts as a soluble ligand to induce angiogenesis, and, while bevacizumab binds VEGF-A, the antibody does not exhibit effector functions.^11^ Binding assays were conducted to characterize the higher order structure of the two antibodies and particularly the Fc domains of ABP 215 and bevacizumab. The neonatal Fc receptor (FcRn) binds to IgG in the Fc region and mediates IgG homeostasis in humans. The results demonstrate that ABP 215 has similar FcRn binding compared with bevacizumab (Table 3). Relative binding to multiple additional Fc gamma receptors with no established role in the efficacy or safety of ABP 215 and bevacizumab (FcγRIa, FcγRIIa, FcγRIIb, FcγRIIIa (158V), FcγRIIIa (158F) and FcγRIIIb) and the first subcomponent of the C1 complex of the classical pathway of complement activation (C1q) were also performed for characterization purposes. The results shown in Table 3 support that ABP 215 has similar relative Fc receptors and C1q binding compared to bevacizumab, thus confirming the minor differences observed in glycan levels did not affect binding activities in the Fc region. Overall, the biological activity results support the conclusion that ABP 215 is highly similar to bevacizumab.

### General properties

Following regulatory agency guidance, biosimilars must demonstrate that the “strength” is the same as the reference product.^16^ The analytical similarity assessment of ABP 215 and bevacizumab for product strength was measured by protein concentration and volume and were found to be similar (Table 2). In addition to the final dosage form strengths, other general properties, including osmolality, pH, appearance, color, and clarity, were also compared, and all results met the pre-specified criteria for analytical similarity.

### Host-cell impurities

Any significant differences of the process-related impurities may have an adverse impact upon safety. The residual host cell proteins (HCP) in ABP 215 and bevacizumab were quantified and characterized by orthogonal methods. The HCP ELISA results using an assay specific to ABP 215 cell line and manufacturing process showed ABP 215 had similarly low levels of residual HCP compared to bevacizumab (Table 2). To further characterize the type of HCPs present in samples, a reverse-phase two-dimensional liquid chromatography (2D-LC) coupled with online mass spectrometry involving data-independent acquisition (2D-LC-MS) method was used to identify and quantify HCPs by mass spectrometry.^17^^,^^18^ Results show no new HCP was present in ABP 215 compared to bevacizumab. Finally, a 2D differential in-gel electrophoresis method comparing samples was performed to confirm the absence of new species in a qualitative manner. In addition, residual protein A and DNA were also in very low levels or below detection in both ABP 215 and bevacizumab.

## Discussion

ABP 215 was developed by Amgen Inc. and has been approved by the FDA and EMA as a biosimilar to bevacizumab. A comprehensive analytical strategy was developed and executed to assess the structural and functional similarity of ABP 215 and bevacizumab. The similarity assessment assessed critical and relevant attributes, including the primary structure, higher order structure, particles and aggregates, product-related substances and impurities, thermal forced degradation, biological activities, general properties, and process-related impurities for both ABP 215 and bevacizumab. The totality of the similarity results demonstrates that ABP 215 is analytically highly similar to bevacizumab. Although some minor differences in physicochemical attributes were observed between ABP 215 and bevacizumab, the biological and functional similarity results show these minor differences do not affect functions relevant to the mechanism of action of ABP 215 and bevacizumab. The results also demonstrate bevacizumab from both US and EU are analytically comparable. Thee analytical similarity assessment results provide the foundation for the scientific justification of extrapolation of the indications approved for bevacizumab to ABP 215.

## Materials and methods

ABP 215 was manufactured by Amgen Inc. US-licensed bevacizumab manufactured by Genentech, Inc. (a Roche company), and EU-authorized bevacizumab manufactured by Roche were procured over a period of approximately 6 years. The reference products were stored and handled according to the manufacturer's instructions and tested as part of the analytical similarity assessment plan. ABP 215 was developed to reflect the same strength and presentations approved in the US and the EU.

This article does not contain any studies with human participants or animals. This paper was prepared according to the ICMJE Uniform Requirements and the International Society for Medical Publication Professionals' “Good Publication Practice for Communicating Company-Sponsored Medical Research: The GPP2 Guidelines.”

### Intact and reduced deglycosylated mass analysis

The molecular masses of intact molecules were determined by electrospray ionization-time of flight-mass spectrometer (ESI-TOF-MS) analysis. Samples were separated from buffer components and introduced to the mass spectrometer by size exclusion chromatography using an Ethylene Bridged Hybrid column. The resulting summed ion spectra were deconvoluted to produce molecular mass profiles. The theoretical mass calculations assumed no C-terminal lysine residues on the HC and that all the disulfides were intact.

The molecular masses of reduced and deglycosylated HC and LC were evaluated using ESI-TOF-MS to provide further assurance the polypeptide compositions were as expected. Samples were treated with PNGase F (Wako Chemicals) to remove N-linked glycans, subsequently denatured with guanidine hydrochloride (Thermo Scientific), and the disulfides reduced with tris (2-carboxyethyl) phosphine hydrochloride (Thermo Scientific). The samples were separated from buffer components and introduced to the mass spectrometer via size exclusion chromatography. Summed ion spectra were deconvoluted to produce molecular mass profiles, which were compared with theoretical mass values.

### Reduced and non-reduced peptide map

Reduced peptide map analysis was conducted by enzymatic digestion with trypsin (Roche Life Science). The sample was treated with dithiothreitol for reduction, sodium iodoacetic acid (IAA, Sigma-Aldrich) for alkylation, and PNGase F for the N linked glycan removal prior to digestion. The resulted peptides were separated by reversed phase UPLC using an increasing gradient of acetonitrile in water. The eluted peaks were detected by UV absorbance (214 nm) and the peptides were identified by on-line mass spectrometry (LC-MS/MS) using hybrid Ion Trap-Orbitrap Mass Spectrometer.

For non-reduced peptide map, samples were digested with trypsin under denaturing, but non-reducing conditions. The resulting peptides were analyzed by RP-HPLC using an increasing gradient of acetonitrile in water with UV detection at 214 nm. The peptides containing disulfide bonds were identified by comparing peptides generated under reducing and non-reducing conditions. Confirmation of peptide identity was achieved using an HPLC coupled with an electrospray mass spectrometer, allowing accurate determination of the molecular mass of each peptide.

### Glycan map

N-linked glycans were evaluated by glycan map analysis using hydrophilic interaction liquid chromatography (HILIC) with fluorescence detection. Glycan mapping involved release of N-glycans through treatment with the amidase PNGase F. The reducing termini of the released glycans were then labeled through reductive amination with a fluorescent tag (2 aminobenzoic acid, 2-AA), and the labeled glycans were separated by HILIC. Peak identification was performed using mass spectrometry by coupling the HILIC HPLC with an ion-trap mass spectrometer for verification against the expected glycan mass.

### Isoelectric points

The pIs were determined by cIEF analysis. cIEF was performed on a capillary electrophoresis separation instrument equipped with a neutral-coated capillary (Beckman Coulter). Samples were electrophoresed through a pH gradient produced by an ampholytic solution (GE Healthcare) until they reached the pH equal to their pI and were then mobilized and detected by UV absorbance (280 nm) as they passed through a detection window in the capillary. The pIs of the sample peaks were determined using a linear regression between two pI marker peaks (Protein Simple).

### Higher order structure by fourier-transform infrared spectroscopy and near ultraviolet-circular dichroism spectroscopy

Secondary structure was assessed by FTIR spectroscopy. The spectrum of the formulation buffer blank was recorded under identical conditions and subtracted from the protein solution spectra. The second derivative spectrum was calculated using a 9-point smoothing of the resulting spectra. Spectral similarity of testing molecule against reference molecule was calculated using the Thermo OMNIC software QC Compare function where 100% similarity indicates identical spectra.

Tertiary structure was assessed by near UV-CD spectroscopy. Protein solutions were diluted to approximately 0.7 mg/mL in test buffer for the near UV-CD measurements using cuvettes with a path length of 1 cm. The spectra were corrected for protein concentration and contributions from the buffer and were reported as CD ellipticity. Spectral similarity was calculated using the Thermo OMNIC software QC Compare function where 100% similarity indicates identical spectra.

### Thermal stability by differential scanning calorimetry

Thermal stability was assessed by DSC using a system in which temperature differences between the testing sample and buffer cells were continuously measured and calibrated. The unfolding of the protein molecules appears as an endothermic transition on the DSC thermogram, and is characterized by the thermal melting temperatures (T_m_). The protein concentration of the testing solutions was approximately 0.5 mg/mL, which was obtained by diluting the solutions in formulation buffer.

### Subvisible particles by light obscuration and micro-flow imaging

Subvisible particles were assessed by light obscuration (LO) using a HIAC 9703+ liquid particle counting system equipped with an HRLD 150 sensor. Particle concentration results were reported as cumulative particle counts per container for ≥ 2, ≥ 5, ≥ 10, and ≥ 25 µm size ranges.

Subvisible particles were also assessed by MFI particle imaging system containing a flow cell and a digital camera. Cumulative particle counts per mL for ≥ 5 μm particles were reported. To quantify product-related particles that are likely proteinaceous and thus have a higher risk for immunogenicity, the MFI data were further analyzed for the concentration of ≥ 5 μm non-spherical particles with an aspect ratio of < 0.85.

### Submicron particles by dynamic light scattering and field flow fractionation

Submicron particles were assessed by dynamic light scattering. Protein solutions were diluted with formulation buffer to approximately 1 mg/mL, and triplicate measurements were taken for each test solution.

Submicron particles were also assessed by FFF. Product solutions were injected neat into the FFF system and UV and light scattering (LS) detectors were used to detect the presence of monomer, aggregates, and submicron particles. Polystyrene particle standards of 100 nm, 200 nm, and 500 nm were used as positive controls, and formulation buffer was used as the negative control.

### Aggregates by analytical ultracentrifugation sedimentation velocity

Aggregates were assessed by AUC-SV. Product solutions were diluted with formulation buffer to approximately 0.5 mg/mL, and the AUC-SV measurements were taken at 45, 000 rpm, with absorbance at 280 nm as detection. Triplicate measurements were taken for each test solution.

### Aggregates by size exclusion-high-performance liquid chromatography with light scattering detection

Aggregates were also assessed by SE-HPLC-LS. The SE-HPLC method used employed an LS detector, a refractive index (RI) detector, and a UV detector at 280 nm. Product solutions were injected neat into the system at a load of approximately 280 µg. For molar mass calculation, an RI increment value of 0.185 mL/g was used. Results were reported as the molar mass of monomer, dimer, and high molecular weight species.

### Size variants by size exclusion-high-performance chromatography, reduced and non-reduced capillary electrophoresis-sodium dodecyl sulfate

Native, or non-denatured, size variants were analyzed by size exclusion-HPLC (SE-HPLC). SE-HPLC measurements were made on an Agilent 1100 HPLC system with a Tosoh Bioscience TSK-GEL G3000SW column. Analytes were monitored by UV absorbance at 280 nm, and purity was evaluated by determining the peak area of each species as a percentage of the total peak area.

Capillary electrophoresis-sodium dodecyl sulfate (CE-SDS) was used for separation of denatured protein size variants under reduced or non-reduced conditions. For non-reduced condition, drug product samples were denatured using sodium dodecyl sulfate at 60^o^C for 5 minutes. For reduced condition, β-mercaptoethanol was added to the protein denaturation step to reduce the disulfide bonds and incubation was performed at 70^o^C for 10 minutes. After denaturation, both reduced and non-reduced samples were injected onto a bare, fused silica capillary and separated based on hydrodynamic size resulting from an applied electric field in which migration time of smaller size proteins is inversely related to overall size. Analytes were monitored by UV absorbance at 220 nm, and purity was evaluated by determining the peak area of each species as a percentage of the total peak area.

### Charge variants by cation exchange-high-performance liquid chromatography

Charged isoforms in drug product samples were separated on a CEX-HPLC Pro Pac WCX-10 analytical column. Eluted fractions using a salt gradient were monitored by UV absorbance at 280 nm and purity was evaluated by determining the peak area of each charged isoform group (acidic, main, and basic peaks) that eluted separately as a percentage of the total peak area.

### Stability and degradation at accelerated conditions

The thermal stability and degradation profiles of ABP 215 drug products and bevacizumab were determined at 25, 40, and 50^o^C. Subsequent characterization of the degradation samples was conducted using SE-HPLC, rCE-SDS, nrCE-SDS, CEX-HPLC, and potency assays.

### Protein concentration

The protein concentration in the solution was determined by UV absorbance using the product extinction coefficient and the density values for both sample and formulation buffer.

### Volume

The analytical procedure for volume determination complies with USP <1>, PhEur 2.9.17, and JP 6.05. It is a quantitative method utilizing the product density and measuring its mass to calculate its volume.

### Host cell impurities

The residual HCP ELISA method was developed specifically for the ABP 215 cell line and process. This method provides quantitative measurement of total HCP detected through the use of a proprietary anti-HCP polyclonal antibody reagent (Amgen).

### Potency by HUVEC proliferation inhibition assay

The proliferation inhibition bioassay is a quantitative cell-based assay utilizing HUVEC (Lonza) that measures the dose-dependent inhibitory effects of ABP 215 or bevacizumab on proliferation of cells expressing VEGFR. HUVEC express VEGFR-1, VEGFR-2 and VEGFR-3 receptors, as well as the co-receptors, neuropilin (NRP)-1 and NRP-2, which, upon interaction with VEGF-A, results in endothelial cell proliferation. In this method, HUVEC were incubated with varying concentrations of ABP 215 reference standard, control, and test samples in the presence of a constant concentration of VEGF-A (R&D Systems, Cat. No. 298-VS). After a timed incubation, an adenosine triphosphate (ATP) specific luminescent reagent (Promega) was added to the assay plates. Addition of this reagent resulted in cell lysis and generation of luminescence signal, which was proportional to the amount of ATP present. The amount of ATP present was directly proportional to the number of viable cells in the culture and inversely proportional to the concentration of tested samples. After assessing parallelism of the dose-response curves, the sample binding relative to the reference standard was determined using a 4-parameter logistic model fit using SoftMax® Pro Software (Molecular Devices). Results were reported as percent relative potency values.

### VEGF-A binding by ELISA and SPR

A solid phase ELISA was used to determine binding to recombinant human VEGF-A. Recombinant VEGF-A (R&D Systems, Cat. No. 293-VE) was coated onto the wells of microtiter ELISA plates. A serial dilution of reference standard, control, and test sample(s) were added and incubated. Following a wash step, a goat anti-human IgG (Fc fragment) conjugated to horseradish peroxidase (HRP; ThermoFisher, Cat. No. 31413) was added to detect bound samples. After a final wash, a substrate/chromogen solution was added to the wells. The substrate changes color in the presence of HRP in proportion to the amount of ABP 215 or bevacizumab bound to VEGF-A. The reaction was stopped with 1.0 M phosphoric acid (Fisher Scientific) and absorbance was measured with a microplate reader. After assessing parallelism of the dose-response curves, the sample binding relative to the reference standard was determined using a 4-parameter logistic model fit using SoftMax® Pro Software (Molecular Devices). Results were reported as percent relative binding values.

The surface plasmon resonance (SPR) analysis was conducted at 25°C using a ProteOn XPR36 optical biosensor (Bio-Rad) equipped with a GLC sensor chip (Bio-Rad). Samples were captured on the GLC chip surface by a goat anti-human IgG1 capture antibody (Jackson ImmunoResearch Laboratories, Cat. No. 109-005-098) and immobilized using standard amine coupling chemistry. Recombinant human VEGF-A (R&D Systems, Cat. No. 298-VS) was injected at concentrations ranging from 50.0 to 3.13 nM and analyzed in triplicate. The data were aligned and double referenced using the ProteOn Manager 3.1.0 version 3.1.06 software (Bio-Rad). The data were then fit using Scrubber v2.0^©^ software (BioLogic), which is an SPR non-linear least squares regression fitting program. Results are reported as the average of three intra-assay replicates per lot. The association and dissociation phases for all VEGF-A concentrations were monitored for 240 seconds each. In addition, a long dissociation phase experiment of 5400 seconds was performed using the 50.0 nM VEGF-A concentration in order to better assess the slow dissociation rate of the antibodies. The binding kinetics were fit using a 1:1 binding model.

### FcRn binding by AlphaScreen

A two-step AlphaScreen® (Perkin Elmer) receptor binding assay was used to quantify the binding of human IgG Fc domain and FcRn. The assay measured the dose-dependent signal decrease observed when an Fc-containing sample is added to a reaction-containing FcRn-His (Amgen) and Fc-biotin (Amgen). Activity was determined by comparing the sample response to the response obtained for the reference standard. The sample binding relative to the reference standard was determined using a 4-parameter logistic model fit using SoftMax® Pro Software (Molecular Devices). Results were reported as percent relative binding values.

### FcγRIa and FcγRIIIa by AlphaLISA

Relative binding to FcγRIa, FcγRIIIa (158V) and FcγRIIIa (158F) was quantified using AlphaLISA® assays (Perkin Elmer). FcγRIa, FcγRIIIa (158V) and FcγRIIIa (158F) GST-fusion proteins were generated at Amgen, Inc. Thousand Oaks, CA, USA. The AlphaLISA® assay is an Amplified Luminescent Proximity Homogenous Assay (Alpha) designed to measure the level of FcγR binding to the Fc portion of IgG1 antibodies. The assay contained two bead types, an acceptor bead and a donor bead. The acceptor bead binds recombinant human FcγR-glutathione-s-transferase (FcγR-GST). The donor beads were coated with a hydrogel that contains phthalocyanine, a photosensitizer, and streptavidin, which binds to biotinylated human IgG1 (Amgen). When bevacizumab or ABP 215 IgG1 is present at sufficient concentrations to inhibit the binding of FcγR-GST to the biotinylated human IgG1, a dose-dependent decrease in emission is observed using a plate reader. The sample binding relative to the reference standard was determined using a 4-parameter logistic model fit using SoftMax® Pro Software (Molecular Devices). Results were reported as percent relative binding values for either FcγRIa, FcγRIIIa (158V) and FcγRIIIa (158F).

### FcγRIIa, FcγRIIb, and FcγRIIIb Binding by SPR

FcγRIIa, FcγRIIb, or FcγRIIIb (R&D Systems) was immobilized through standard amine coupling chemistry to a BiaCore CM5 chip (GE Healthcare Bio-Sciences). Binding data was collected 4 seconds prior to the end of injection for each concentration of a duplicate sample dilution series. The K_D_ was determined using the steady state affinity model. The binding data generated from the assay run was used to determine the relative binding activity of the test samples in comparison to the reference standard by dividing the K_D_ of the sample by that of the reference standard. Binding is reported as relative binding for FcγRIIa, FcγRIIb, and FcγRIIIb,

### C1q binding by ELISA

A direct binding ELISA method was developed to assess the binding of ABP 215 to C1q. In this assay, bevacizumab or ABP 215 was adsorbed to a microtiter plate and incubated with C1q (Sigma-Aldrich, Cat. No. C1740). Bound C1q was detected with an anti-C1q-HRP conjugated antibody (Bio-Rad Hercules, Cat. No. 2221–5004P). After a final wash, a substrate/chromogen solution was added to the wells. The substrate changes color in the presence of HRP in proportion to the amount of C1q bound to ABP 215 or bevacizumab. The reaction was stopped with sulfuric acid (Sigma-Aldrich) and absorbance was measured with a microplate reader. The sample binding relative to the reference standard was determined using a 4-parameter logistic model fit using SoftMax® Pro Software (Molecular Devices). Results were reported as percent relative binding values for C1q.

### Inhibition of VEGFR2 RTK autophosphorylation

HUVEC were incubated with varying concentrations of samples in the presence of a constant concentration of VEGF-A (R&D Systems, Cat. No. 298-VS-025/CF). After a timed incubation, the cells were lysed. VEGFR-2 was captured from the lysate onto streptavidin-coated Mesoscale Discovery (MSD, Cat. No. L15SA) plates using a biotinylated antibody against the extracellular portion of VEGFR-2 (R&D Systems, Cat. No. 05–321). This was followed by the addition of a murine anti-phosphotyrosine monoclonal antibody (EMD Millipore, Cat. No. 05–321) for detection of tyrosine phosphorylation on the captured VEGFR-2 and an anti-murine IgG conjugated with ruthenium (MSD, Cat. No. R32AC-1) for signal generation. The addition of a tripropylamine-containing buffer (MSD, Cat. No. R92TC-2) followed by electrical activation of the plate resulted in an electrochemiluminescent (ECL) signal detected by a plate reader. The ECL signal counts were proportional to the level of VEGFR-2 tyrosine phosphorylation.
